# Comparing population and incident data for optimal air ambulance base locations in Norway

**DOI:** 10.1186/s13049-018-0511-4

**Published:** 2018-05-24

**Authors:** Jo Røislien, Pieter L. van den Berg, Thomas Lindner, Erik Zakariassen, Oddvar Uleberg, Karen Aardal, J. Theresia van Essen

**Affiliations:** 1 Faculty of Health Sciences, University of Stavanger, Stavanger, Norway; 2 Norwegian Air Ambulance Foundation, Department of Research, Drøbak, Norway; 3 Rotterdam School of Management, Erasmus University, Rotterdam, the Netherlands; 4 Stavanger Acute medicine Foundation for Education and Research (SAFER), Stavanger, Norway; 5 Department of Global Public Health and Primary Care, University of Bergen, Bergen, Norway; 6 Department of Emergency Medicine and Pre-Hospital Services, St.Olav’s University Hospital, Trondheim, Norway; 7 Delft Institute of Applied Mathematics, Delft University of Technology, Delft, the Netherlands; 8 Centrum Wiskunde & Information, Amsterdam, the Netherlands

**Keywords:** HEMS, Air ambulance, Facility location problem, MCLP, Population density, Incidents, Coverage

## Abstract

**Background:**

Helicopter emergency medical services are important in many health care systems. Norway has a nationwide physician manned air ambulance service servicing a country with large geographical variations in population density and incident frequencies. The aim of the study was to compare optimal air ambulance base locations using both population and incident data.

**Methods:**

We used municipality population and incident data for Norway from 2015. The 428 municipalities had a median (5–95 percentile) of 4675 (940–36,264) inhabitants and 10 (2–38) incidents. Optimal helicopter base locations were estimated using the Maximal Covering Location Problem (MCLP) optimization model, exploring the number and location of bases needed to cover various fractions of the population for time thresholds 30 and 45 min, in green field scenarios and conditioned on the existing base structure.

**Results:**

The existing bases covered 96.90% of the population and 91.86% of the incidents for time threshold 45 min. Correlation between municipality population and incident frequencies was −0.0027, and optimal base locations varied markedly between the two data types, particularly when lowering the target time. The optimal solution using population density data put focus on the greater Oslo area, where one third of Norwegians live, while using incident data put focus on low population high incident areas, such as northern Norway and winter sport resorts.

**Conclusion:**

Using population density data as a proxy for incident frequency is not recommended, as the two data types lead to different optimal base locations. Lowering the target time increases the sensitivity to choice of data.

## Background

Helicopter emergency medical services (HEMS) are common in many health care systems in the developed world [1, 2]. Though empirical studies show evidence both in favor of the service [3–5] and not [3, 6, 7], HEMS are expanding throughout the world, bringing advanced medical care, treatment options and decision making competence to the scene, shortening transport time and providing access to remote areas [8–10].

A paramount principle in Norwegian health legislation is that all citizens should have equal access to publicly funded health care regardless of their residential pattern [11]. In Norway, HEMS is considered essential in order to achieve the desired equality in access to health care. Despite large geographical distances and substantial uninhabited areas, the government requirements state that 90% of the population should be reached by a physician manned ambulance service on scene within 45 min [12]. This national requirement does not necessarily imply a HEMS doctor, but refers to any doctor involved in the out of hospital emergency care. A distinct feature of the Norwegian healthcare system is the important function of the general practitioner, which is considered the ‘gatekeeper’ of the Norwegian Health Care system [13]. The objective of the Norwegian air ambulance service, a public nationwide anaesthesiologist manned air ambulance service, is to provide advanced emergency medicine to critically ill or severely injured patients. About 70% of the missions are medical, while 30% is trauma [14]. The service operates 24/7/365.

In order to ensure optimal coverage, the location of the air ambulance bases is crucial. Currently, there are 12 helicopter ambulance bases in Norway with 13 helicopters providing HEMS, established gradually through historical local engagement from the late 1970s [15]. Academic literature on the topic is scarce [16], but a recent study indicates that using a more mathematically rigorous approach to base location optimization suggests different base locations than the existing ones [17].

For any emergency medical service (EMS), it is important to locate vehicles in such a way that incidents can be served as quickly as possible. Various mathematical models tackle this problem, such as the Maximal Covering Location Problem (MCLP) [18]. The MCLP maximizes the weighted number of demand locations covered within a desired service distance, or time, from a facility by allocating a fixed number of facilities. Conversely, the model allows for the determination of the least number of bases needed in order to guarantee a certain pre-specified coverage.

Norwegian government regulations are with respect to population coverage. But whether population data is a reasonable proxy for actual incidents for which HEMS is needed is unknown. In Norway, there is large variation in the number of municipality incidents per 1000 inhabitants, with the higher ratios in Northern Norway, where the population density is the lowest [11]. A reasonably high correlation between population density and incident frequency thus cannot immediately be assumed. Also, in Norway weather varies strongly throughout the year, affecting where Norwegians spend their time: in winter many Norwegians find their way to the snow covered mountains, while during summer they spend time by the coast. Seasonal variations in the number of trauma admissions has been demonstrated in several international studies [19, 20].

The MCLP is generally regarded as a robust method for locating emergency vehicles, but if the underlying data does not properly represent the situation under study, results might still be unreliable. The aim of the present study was to compare the optimal locations of air ambulance bases using the MCLP model on both population density and incident frequency data for all municipalities in Norway. We performed both green field analyses, assuming clean slate, and optimization conditioned on the existing base structure.

## Methods

### Data material

Mainland Norway covers 323,780 km^2^ at the far North of Europe, stretching 1790 km from north to south. Official population statistics are freely available from Statistics Norway [21]. January 1st 2015, the population in Norway was 5.2 million [22]. The country has a mixed rural and urban population with county population density ranging from 1129.5 inh/km^2^ in Oslo to 1.5 inh/km^2^ in Finnmark.

A previous study has shown that differences between using municipality level population data and fine grid data are negligible [17]. Municipality data has the benefit of reducing computation times considerably and was used in this study. In 2015, Norway consisted of 428 municipalities with a median (5–95 percentile) of 4675 (940–36,264) inhabitants. For each municipality, there is a population weighted centroid representing the population centre of the municipality.

Aggregated yearly municipality incident data for primary acute missions are available from the National Air Ambulance Services upon request. In 2015, the number of incidents had a median (5–95 percentile) of 10 (2–38). About 70% of the missions are medical, 30% are trauma [14]. Called off cases are not included, be it due to no medical need, technicalities, or concurrent tasking.

Air ambulances in Norway are allowed a 15 min pre-flight preparation time, but the average of HEMS in Norway is 5.5 min [11] and this latter number was used in the calculations. Helicopter ground speed depends on wind direction and strength. In the mathematical models, we used 220 km/h, as an overall average number, taking into account the different helicopter types and the helicopter speeds used during each mission (take off, cruise phase, and landing phase including identification of suitable landing sites).

### Methods

Optimal base locations were determined by modelling the problem as a Maximal Covering Location Problem (MCLP) [18]. The MCLP model maximizes the number of demand locations covered by at least one ambulance, weighted by the number of inhabitants or accumulated yearly incidents in each demand location. That is, it maximizes the number of inhabitants or incidents covered within a desired time by optimal allocation of a pre-defined fixed number of facilities. Conversely, the model can be used to determine the least number of bases needed in order to guarantee a certain coverage of the population or incidents. In the MCLP model it is assumed that an ambulance is always available at a base whenever needed.

For analyses of both population data and incident frequency data, we used all of the 428 municipalities as both potential base locations and demand locations. The travel times, including a 5.5-min fixed pre-flight preparation time, from all potential base locations to all demand locations was then calculated, that is, *from* all *to* all municipalities, and optimal base locations determined.

To explore the practical consequences of various target times, we calculated the number of bases needed to cover various percentages of the population, and incidents, for threshold times 45 and 30 min.

Using both municipality population and incident frequency data we first computed the optimal base locations assuming no bases existed, so-called green field analysis. As such an analysis is rarely practically feasible, we also performed conditional optimization: given the existing 12 bases in Norway in the beginning 2015 what would be the additional gain of relocating or adding one base.

The models are implemented in Java and solved with IBM ILOG CPLEX Optimization Studio (CPLEX 12.6.2).

## Results

**Fig. 1:**
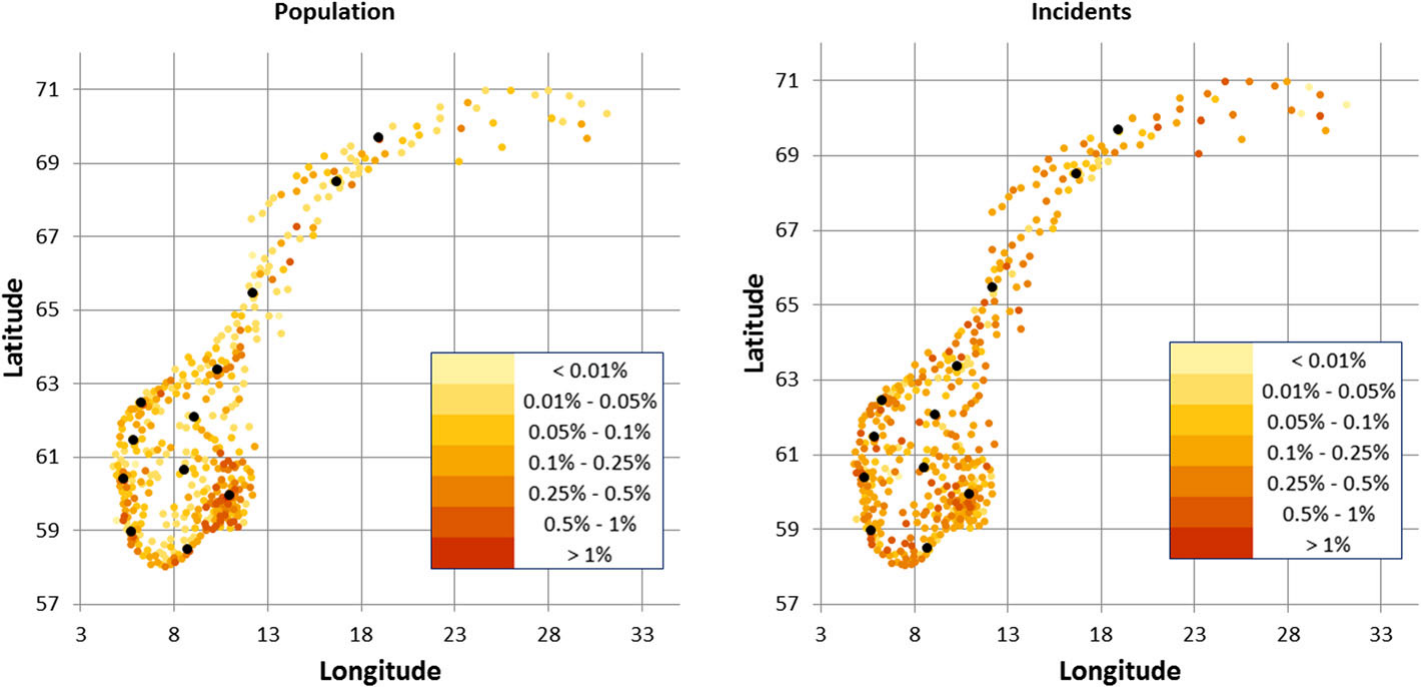
Population density heat map of Norway (left) and incident frequencies (right). Colour dots represent centroid location of the 428 municipalities. The 12 existing air ambulance bases superimposed

**Fig. 2:**
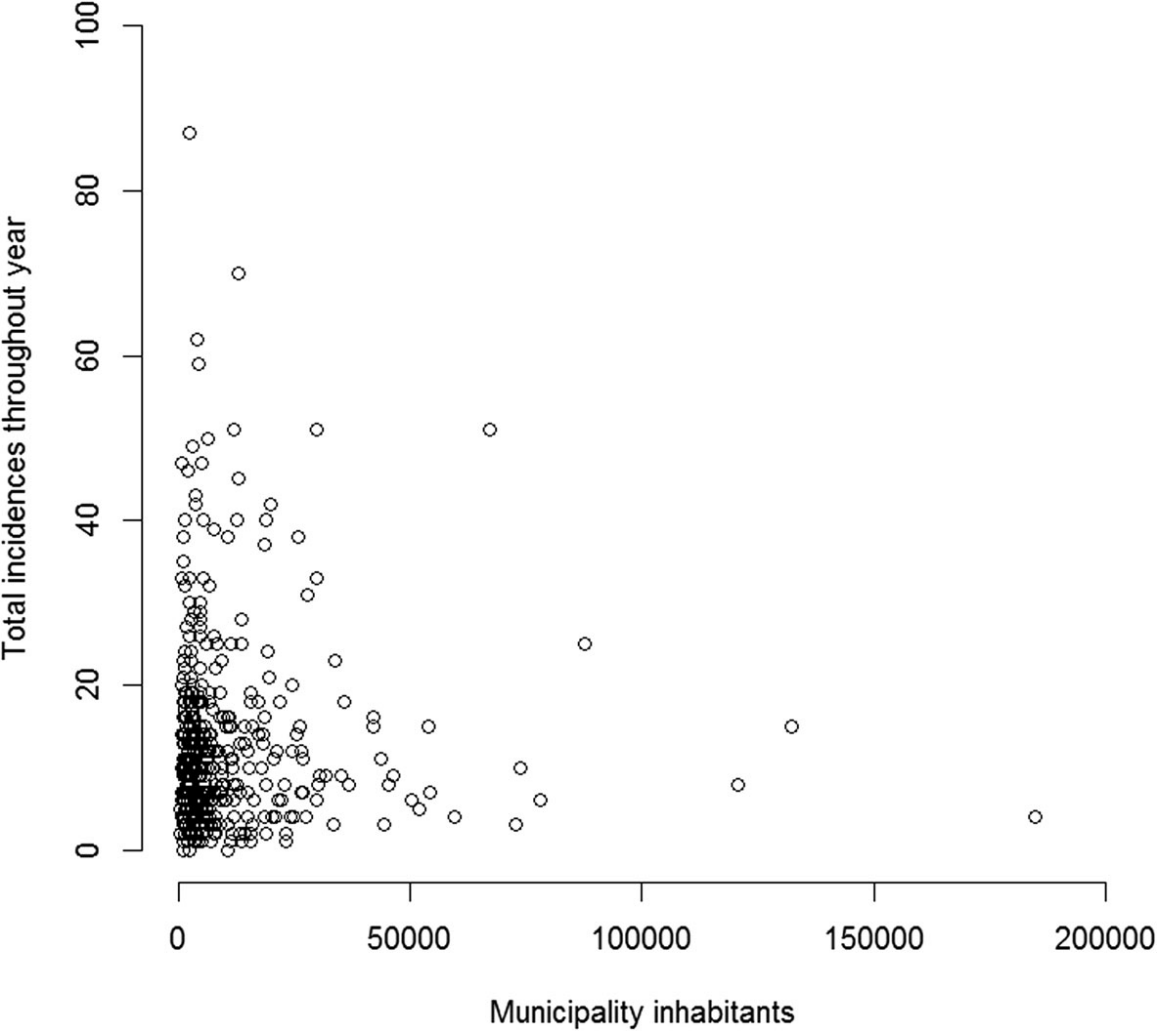
Municipality population vs total number of municipality incidents in Norway 2015

Municipality population density and incident frequency maps are shown in Fig. 1. Municipality population size and yearly number of incidents was uncorrelated, with a Spearman’s rho of −0.0027 (Fig. 2).

### Optimization in green field scenarios

The relationship between the number of bases and coverage for various target times based on either population or incident data is summarized in Table 1.

**Table 1:** Coverage using population density or aggregated municipality incidence data, for various HEMS target times, in greenfield scenario

Data used	Time threshold	Target coverage	Number of bases needed	Percentage of population covered	Percentage of incidents covered
Population	45	90%	5	93.32	78.31
Population	45	95%	6	96.29	83.55
Population	45	100%	10	100.00	100.00
Incidents	45	90%	7	96.18	94.73
Incidents	45	95%	8	98.22	97.91
Incidents	45	100%	10	100.00	100.00
Population	30	90%	9	91.97	69.30
Population	30	95%	12	96.36	81.88
Population	30	100%	22	100.00	100.00
Incidents	30	90%	14	93.81	92.04
Incidents	30	95%	16	94.76	96.27
Incidents	30	100%	22	100.00	100.00

**Fig. 3:**
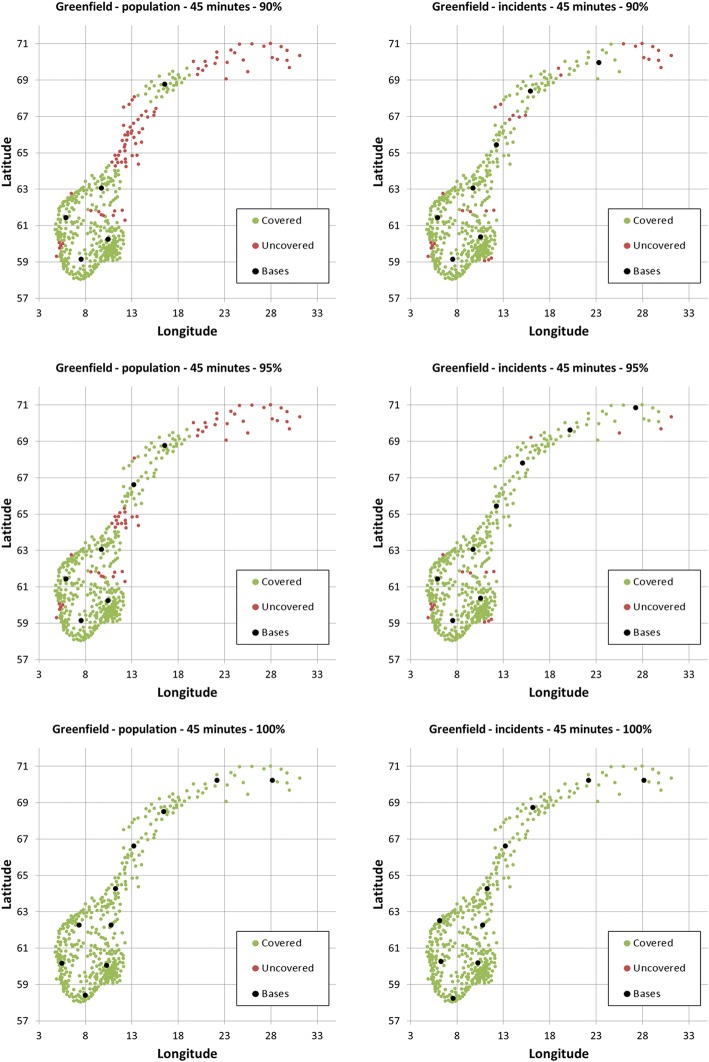
Optimal location to achieve 90, 95 and 100% coverage within 45 min based on municipality population data (left column) and aggregated yearly number of municipality incidents (right column) in Norway in 2015

With a threshold of 45 min, 90% of the population could be covered using five bases, while seven bases are needed to cover 90% of the incidents. With six optimally located bases one could cover 95% of the population, while eight bases are needed to cover 95% of the incidents. A total of 10 bases is needed to cover the whole population, or similarly all incidents. The corresponding optimal base locations are shown in Fig. 3. While base locations are fairly similar using either population or incident data in the southern part of Norway, the two data types indicate different optimal base locations in the northern part of Norway.

**Fig. 4:**
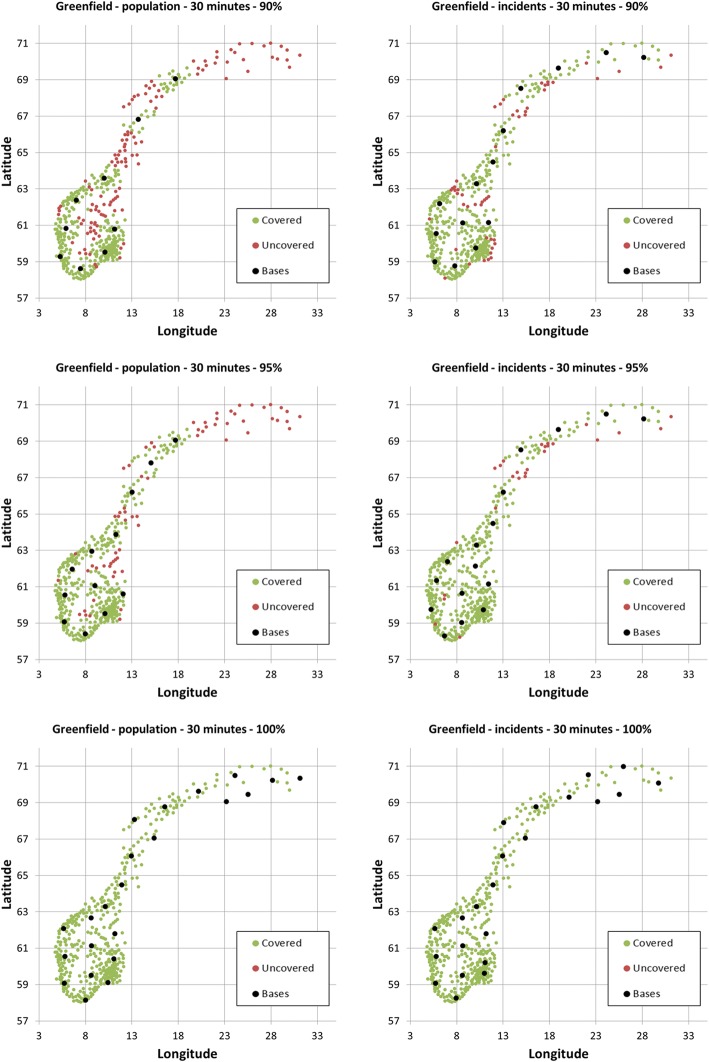
Optimal location to achieve 90, 95 and 99% coverage within 30 min based on municipality population data (left column) and aggregated yearly number of municipality incidents (right column)

Decreasing the threshold to 30 min substantially increases the number of bases needed to achieve the same coverage. Nine bases are needed to cover 90% of the population and 14 bases to cover 90% of the incidents, while 12 bases are needed to cover 95% of the population and 16 bases to cover 95% of the incidences. In order to cover the whole population, one would need 22 bases, and also 22 to cover all incidents. Corresponding base locations are shown in Fig. 4. Using actual incidents rather than population data implies two main changes in optimal base locations. Firstly, the need for more bases in Northern Norway, where population is scarce but with comparably more incidents where HEMS is needed. Secondly, a re-arrangement of the bases in the Oslo region, where about one third of Norwegians live.

### Optimization conditioned on existing base structure

**Fig. 5:**
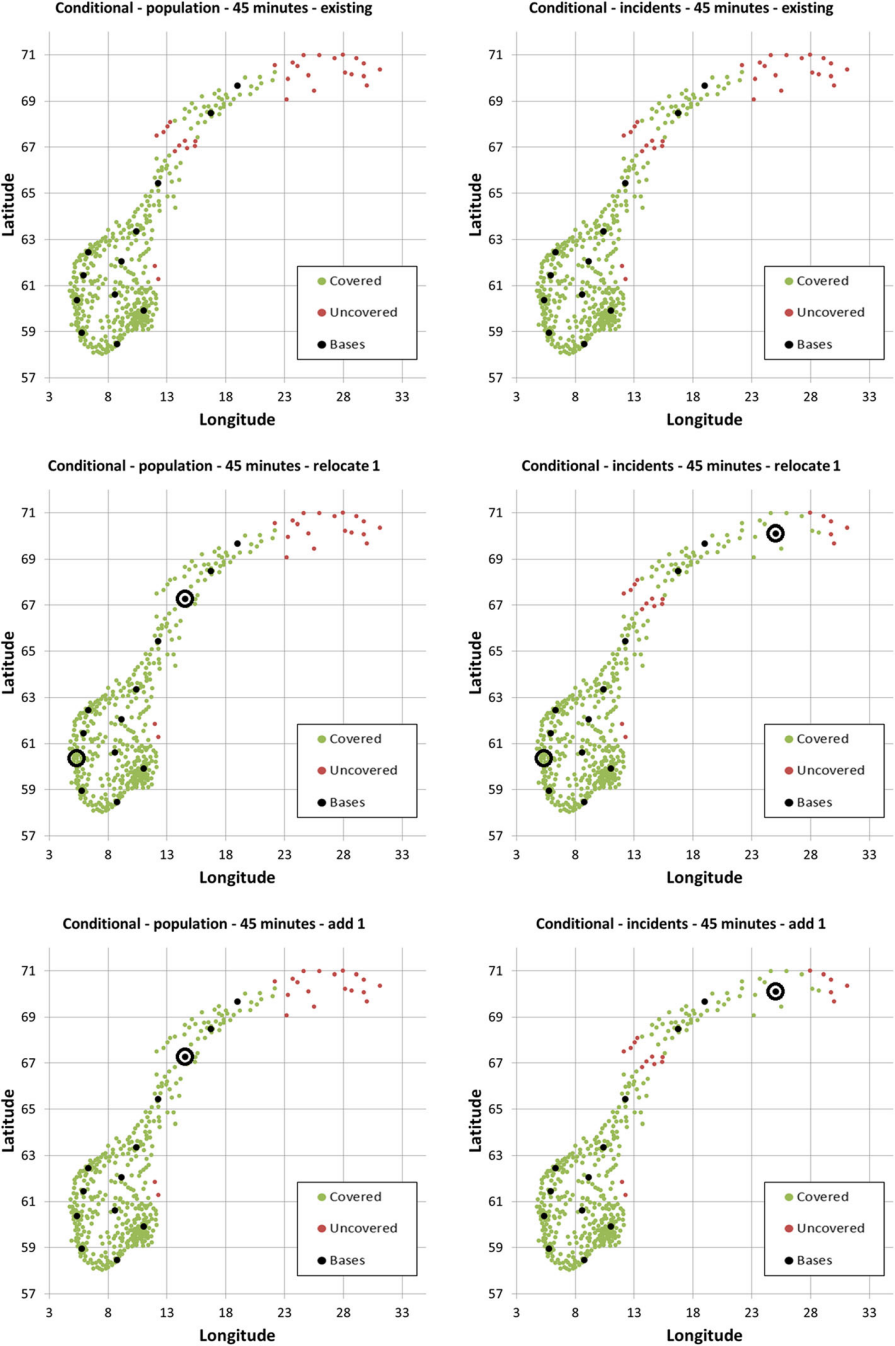
Optimal HEMS base locations when relocating or adding one base to the existing base structure, based on population data (left column) and incidence data (right column) for a 45 min threshold

Using a 45 min threshold, the 12 existing bases cover an estimated 96.90% of the population and 91.86% of the incidents (Table 2). Relocating the Bergen base in western Norway to the northern part of the country, or simply adding a base at this northern location, would increase population and incident coverage. The optimal location of this new northern base depends on whether using population or incident data, with incident data putting the base further to the north, into a less densely populated area (Fig. 5).

**Table 2:** Coverage using population density or municipality incident data, for various HEMS target times, in conditional optimization, based on the existing base structure

Data used	Time threshold	Scenario	Percentage of population covered	Percentage of incidents covered
Population	45	Existing	96.90	91.86
Population	45	Relocate one base	98.40	93.44
Population	45	Add one base	98.40	93.44
Incidents	45	Existing	96.90	91.86
Incidents	45	Relocate one base	97.90	96.35
Incidents	45	Add one base	97.90	96.35
Population	30	Existing	84.70	72.13
Population	30	Relocate one base	87.93	71.50
Population	30	Add one base	88.98	75.35
Incidents	30	Existing	84.70	72.13
Incidents	30	Relocate one base	86.00	74.77
Incidents	30	Add one base	85.68	76.62

**Fig. 6:**
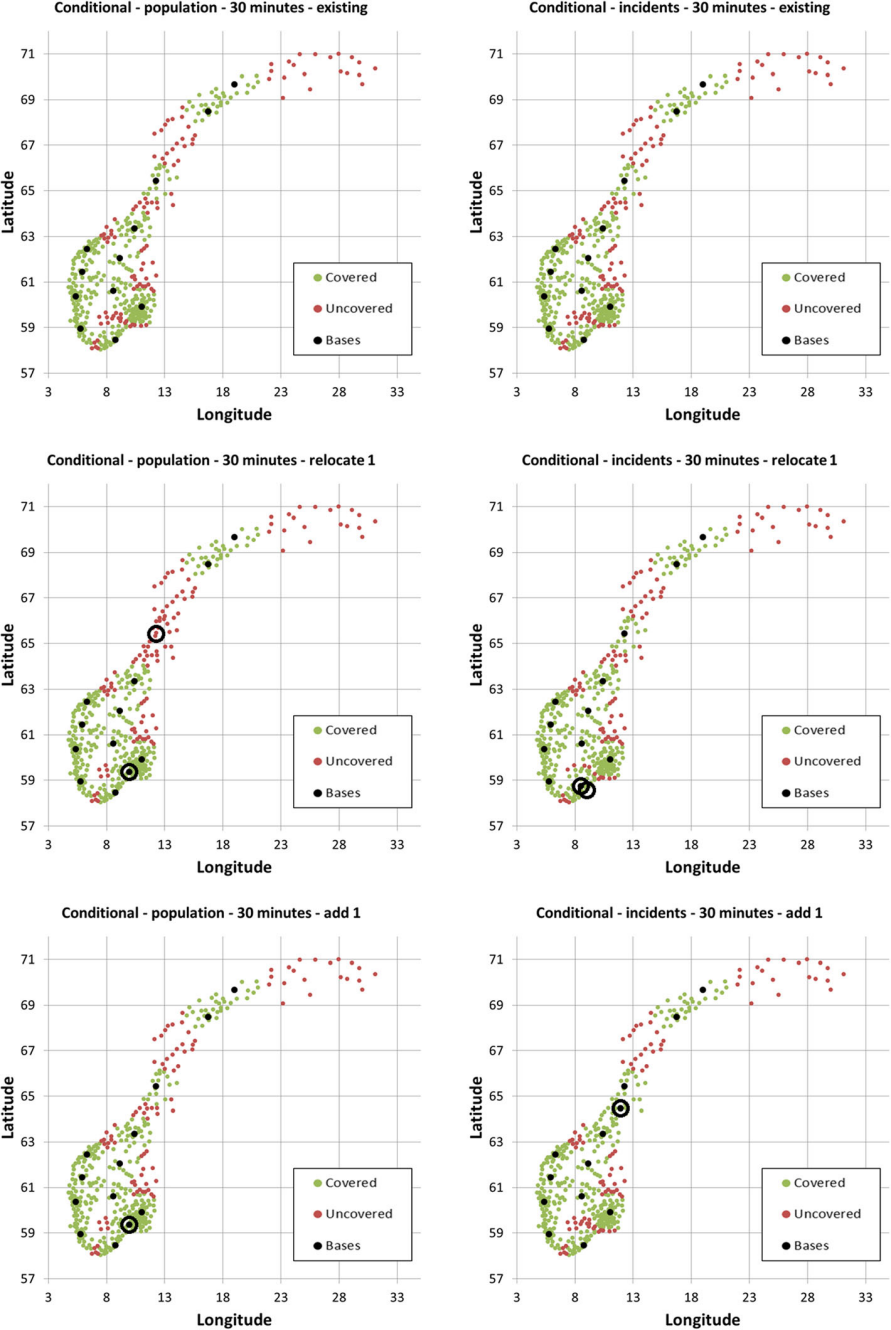
Optimal HEMS base locations when relocating or adding one base to the existing base structure, based on population data (left column) and incidence data (right column) for a 30 min threshold

Changing to a 30 min threshold, the 12 existing bases cover an estimated 84.70% of the population and 72.13% of the incidents (Table 2). Using population data, the Brønnøysund base in the middle of Norway is the least contributive, and coverage increases when relocating the Brønnøysund base to the Oslo region, or simply by adding a base at this new location (Fig. 6). Using incident data paints a different picture. Coverage will increase if moving the Arendal base in southern Norway somewhat further away from the coast, into the mountains, or by adding a new base north of Trondheim in the middle part of Norway (Fig. 6).

## Discussion

Where people live and where they might need immediate medical assistance does not overlap perfectly. Our calculations show that given a target time of 45 min the existing base structure covers 96.90% of the Norwegian population and 91.86% of incidents where HEMS is needed, and that 100% is within reach with moderate adjustments. Which relocations and additions are needed to achieve full coverage does however depend on whether population or incident data is being used in the calculations.

Current Government goals state that 90% of the population should be reached by an ambulance staffed with a medical doctor within 45 min [12]. This statement indirectly assumes that population density is a reasonable proxy for incident frequency, but our present analyses demonstrate how the weak correlation between the two leads to different optimal base locations.

Given the increasing evidence that time is of the essence in pre-hospital medical care [23–26], decreasing target time might be both a political and medical goal for improved health care. Our calculations indicate that lowering the target time seems to increase the impact of choice of data. Lowering the target threshold from 45 to 30 min not only markedly increases the number of bases needed, but also highlights the impact of choice of data. With a 30 min threshold, using population data in the calculations, increased coverage can be obtained by relocating a base from the scarcely populated northern part of Norway to the Oslo region, where about one third of Norwegians live. Using incident data, however, increased coverage can be achieved by relocating a base from the southern city of Arendal to the vicinity of a nearby winter sport location: a place where few people live but many spend their leisure time.

Norway covers a large geographical area with diverse nature and strong seasonal and weather effects. Many Norwegians spend time in the snowy mountains during winter, and by the coast in the summer. These dynamics are already being incorporated into the existing base structure and utilization of available resources, with provisory seasonal bases. Notably, the location of the provisory base at Hovden coincides largely with the optimal relocation of the Arendal base when shifting focus from population to incident data.

The large urban-rural differences in Norway constitute a challenge for the desired equality in health care in Norway. The population density of 1.5 inh/km^2^ in the county of Finnmark in northern Norway, covering one fifth of the Norwegian land area, is low. Any model based on postal address will consequently tend to downplay the importance of the northern part of Norway in terms of base location cost efficiency. Northern Norway does however also have comparably more incidents, as do several of the municipalities with low inhabitant numbers (Fig. 2).

However, while population data are fairly stable, the same does not hold for incident data. The yearly reports on HEMS missions indicate that overall very few missions are not completed, be it due to concurrent events or other, but studies have shown that for certain patient groups a large proportion of eligible patients do not receive a physician team response [27]. Also, ground ambulance HEMS missions are not included, which might affect in particular areas in and around the larger cities. Estimates using incident data are consequently more uncertain than those using population data. Also, this study uses incident data from one year only. Future studies should include studies on multiple years to help establish the uncertainty in the estimates using actual incident response data.

Numerous emergency vehicle location models have been proposed [28, 29]. The MCLP model used in this study assumes that a vehicle is always available at a base location whenever needed. In practice, this assumption will often be overly optimistic, and results from MCLP model thus represent a best-case-scenario. The less valid the assumption, the more vehicles are needed. Adding more vehicles might also change optimal base locations. Simultaneity conflicts in the Norwegian HEMS is a repeating topic in the public discourse regarding HEMS, but to what extent this busy fraction, that is, the proportion of times an emergency vehicle is busy whenever needed due to concurrent tasks, might affect the number of vehicles needed to achieve the desired coverage in the Norwegian HEMS, and the corresponding base locations is difficult to estimate, and has not been explored by the scientific community.

The busy fraction has been taken into account in mathematical models like the Maximum Expected Covering Location Problem (MEXCLP) [30] and the Maximum Availability Location Problem (MALP) [31]. However, Norway’s large urban-rural differences, with population density and yearly incidents varying strongly among municipalities, the busy fraction modelling must be handled with care. Existing models might not be sufficient to properly handle the heterogeneity implied by the large rural-urban differences in geography. Future research should explore this topic.

In the present study, we have not included ground services in the model. It is however unlikely that this would affect optimal air ambulance base locations. The ground service constitutes the backbone of the Norwegian EMS, but the two services represent different levels of care: While the ground service is manned with paramedics, the HEMS is manned with physicians. The two systems are developed not to compete but to complement one another.

Fine detail information on incident locations is currently not available in Norway, but recent research has demonstrated that results using municipality level population data are almost identical to results when using fine detail population data [17]. Municipality level incident data should thus be sufficient for proper analysis, and also has the advantage of being markedly less computer intensive.

As HEMS are expanding throughout the world, knowledge on how to locate the air ambulance bases in order to optimize coverage given the available resources and government goals is of increasing importance. The present analysis demonstrates not only the power of mathematical modelling to answer this question, but the importance of basing optimization on the proper data. Focusing on the population rather than actual incidents might result in a base structure that covers the locations where people receive their mail, but not where they actually need immediate medical assistance.

## Conclusion

Air ambulance systems are an integral part of many health care systems, and in order to achieve optimal population coverage in health care base locations are central. As existing bases are often located based on local historic engagement mathematical modelling can be valuable when building new, or adjusting existing, base structures in order to ensure optimal coverage. Using actual incident data rather than population density data results in different optimal base locations, and the use of population data as a proxy for incident data is thus not recommended. The difference in the optimal number of bases and base locations between population and incident data seems to increase with lower target times.

## Abbreviations

EMS: Emergency medical service
HEMS: Helicopter emergency medical services
MALP: Maximum Availability Location Problem
MCLP: Maximal Covering Location Problem (MCLP)
MEXCLP: Maximum Expected Covering Location Problem
